# Camptothecin exhibits topoisomerase1-independent KMT1A suppression and myogenic differentiation in alveolar rhabdomyosarcoma cells

**DOI:** 10.18632/oncotarget.25376

**Published:** 2018-05-25

**Authors:** David W. Wolff, Min-Hyung Lee, Mathivanan Jothi, Munmun Mal, Fengzhi Li, Asoke K. Mal

**Affiliations:** ^1^ Department of Cell Stress Biology, Roswell Park Cancer Institute, Buffalo, NY 14263, USA; ^2^ Current address: Division of Biotechnology Review and Research IV, Office of Biotechnology Products, Center for Drug Evaluation and Research, Food and Drug Administration, Silver Spring, MD 20993, USA; ^3^ Current address: Department of Human Genetics, National Institute of Mental Health and Neurosciences, Bengaluru, KA 560029, India; ^4^ Department of Pharmacology and Therapeutics, Roswell Park Cancer Institute, Buffalo, NY 14263, USA

**Keywords:** camptothecin, rhabdomyosarcoma, methyltransferase, myogenesis

## Abstract

Alveolar rhabdomyosarcoma (aRMS) is an aggressive subtype of the most common soft tissue cancer in children. A hallmark of aRMS tumors is incomplete myogenic differentiation despite expression of master myogenic regulators such as MyoD. We previously reported that histone methyltransferase KMT1A suppresses MyoD function to maintain an undifferentiated state in aRMS cells, and that loss of KMT1A is sufficient to induce differentiation and suppress malignant phenotypes in these cells. Here, we develop a chemical compound screening approach using MyoD-responsive luciferase reporter myoblast cells to identify compounds that alleviate suppression of MyoD-mediated differentiation by KMT1A. A screen of pharmacological compounds yielded the topoisomerase I (TOP1) poison camptothecin (CPT) as the strongest hit in our assay system. Furthermore, treatment of aRMS cells with clinically relevant CPT derivative irinotecan restores MyoD function, and myogenic differentiation *in vitro* and in a xenograft model. This differentiated phenotype was associated with downregulation of the KMT1A protein. Remarkably, loss of KMT1A in CPT-treated cells occurs independently of its well-known anti-TOP1 mechanism. We further demonstrate that CPT can directly inhibit KMT1A activity *in vitro*. Collectively, these findings uncover a novel function of CPT that downregulates KMT1A independently of CPT-mediated TOP1 inhibition and permits differentiation of aRMS cells.

## INTRODUCTION

Rhabdomyosarcoma (RMS) is the most common soft tissue cancer in children and adolescents [1]. Pediatric RMS has two primary subtypes: embryonal (eRMS) and alveolar (aRMS). Although both are diagnosed based on expression of skeletal muscle markers, eRMS and aRMS have differing gene expression, genetics, and patient outcomes [2, 3]. Approximately 60% of aRMS tumors harbor the PAX3-FOXO1 fusion oncoprotein resulting from a recurrent t(2;13) chromosome translocation [4, 5]. Although approximately 60% of pediatric RMS patients achieve long term survival with current multimodal treatments, more than 70% of those diagnosed with fusion-positive aRMS tumors succumb to their disease [6]. Therefore, there is an immediate need to improve upon the current standard of care and provide better treatment outcomes for patients with PAX3-FOXO1-positive disease using novel therapeutic approaches [2].

A key feature of RMS is the maintenance of an undifferentiated myogenic state [7, 8]. The differentiation-promoting myogenic transcription factor MyoD is expressed in RMS tumors [7, 9], but several studies have demonstrated that its activity for inducing differentiation is compromised through various mechanisms [8]. Numerous studies including ours have demonstrated that activation of MyoD in RMS cells suppresses malignant phenotypes and induces terminal myogenic differentiation [10–13]. Thus, identifying means to modulate MyoD activity of RMS could provide novel therapeutic strategies. We previously reported that histone methyltransferase KMT1A (also known as SUV39H1) is a negative regulator of MyoD in PAX3-FOXO1-positive aRMS [10]. KMT1A represses myogenic differentiation by binding with MyoD at myogenic loci and inhibiting gene transcription by catalyzing trimethylation of lysine 9 on histone 3 (H3K9me3) [10, 14], a transcriptional repressive mark [15, 16]. Indeed, knockdown of KMT1A leads to upregulation of MyoD target genes, including the critical terminal differentiation-promoting myogenic factor MYOGENIN (MyoG) [17]. This leads to terminal myogenic differentiation of aRMS cells and suppression of malignant phenotypes, suggesting that pharmacological targeting of KMT1A may overcome the differentiation blockade in aRMS.

Based on these findings, we sought to identify small molecules which overcome KMT1A-mediated repression of MyoD. A cell-based high throughput screen of pharmacological compounds found the most active primary hit to be the well-established Topoisomerase I (TOP1) poison camptothecin (CPT) [18], which was subjected to further study. We uncovered that treatment with CPT or CPT derivatives promotes MyoD activity and phenotypic terminal muscle differentiation in aRMS cells and in an *in vivo* xenograft model. Furthermore, we found that CPT treatment results in downregulation of the KMT1A protein, and provide compelling evidence that this loss occurs independently of DNA damaging TOP1-DNA cleavage complexes. Finally, we show that CPT directly inhibits the histone methyltransferase activity of KMT1A *in vitro*. Together, our data reveal a novel mechanism by which CPT downregulates KMT1A protein in cells, resulting in the restoration of MyoD function and promoting myogenic differentiation in aRMS.

## RESULTS

### Cell-based screen of a small molecule library uncovers camptothecin as a MyoD activator overcoming KMT1A-mediated repression in myoblasts

**Figure 1:**
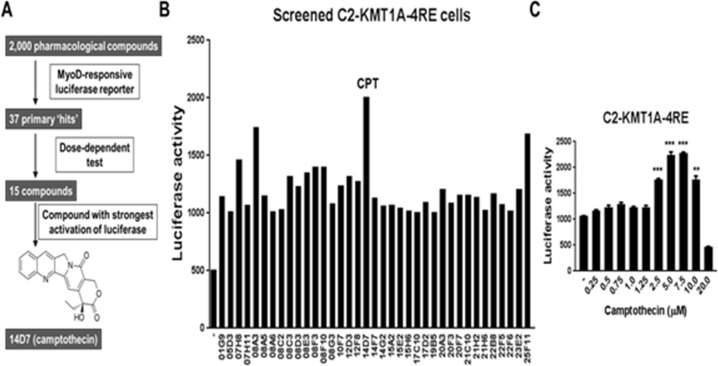
Cell-based screening of a small molecule library identifies camptothecin (CPT) as a potent activator of MyoD in KMT1A-overexpressing myoblast cells **(A)** Diagram depicting the drug screen process which identified CPT as the strongest MyoD activator in C2-KMT1A-4RE reporter myoblast cells from a library of 2,000 compounds. Cells were treated for 36 hours in DM. **(B)** Luciferase activity of 37 initial hit compounds and DMSO control (-) as measured in the primary screen. The compound with the strongest activation of luciferase, camptothecin (14D7), is denoted CPT. **(C)** C2-KMT1A-4RE reporter cells were plated and treated with indicated concentrations of CPT, with DMSO (-) as vehicle control. After 36 hours of treatment in DM, luciferase activity was determined. Error bars represent ±SEM from biological replicates (n=3). ^**^ indicates P<0.01; ^***^ indicates P<0.001 relative to DMSO control.

Our previous study demonstrated that upregulated KMT1A suppresses MyoD activity to block differentiation in aRMS cells [10]. Thus, we performed a cell-based chemical screen of 2,000 pharmacologically active compounds to identify those that activate MyoD in a setting with elevated KMT1A levels. The screen was performed in previously described KMT1A-overexpressing C2C12 murine myoblast cells harboring a MyoD-responsive luciferase reporter gene (C2-KMT1A-4RE) cultured in differentiation media (DM) [19]. In these reporter cells, KMT1A specifically represses MyoD-responsive 4RE luciferase reporter activity. Thus, this screen allowed the identification of compounds which overcome KMT1A-mediated repression of MyoD (Figure 1A). The primary screen identified 37 “hit” compounds that were selected based on a ≥2 fold induction of luciferase activity (Figure 1B). Among the confirmed luciferase-activators, 14D7, the TOP1 poison CPT, showed the strongest activation of luciferase activity (Figure 1B). These primary hits were also subjected to secondary screening in a dose-dependent assay, and 3 of the 15 luciferase activators confirmed following this secondary screening were CPT derivatives (Supplementary Table 1). Due to these findings and the use of CPT derivatives in RMS treatment [20–22], we selected CPT for further study. Thus, CPT was evaluated in an extensive dose-dependent assay of MyoD-responsive luciferase activity in these reporter cells. The results show that CPT induces luciferase activity in a dose-dependent manner, before suppressing it at a highly cytotoxic dose (20 μM) (Figure 1C). Together, these results demonstrate that CPT can overcome KMT1A-mediated repression of MyoD in C2C12 myoblast cells.

### CPT treatment restores MyoD-mediated gene transcription in aRMS cells

**Figure 2:**
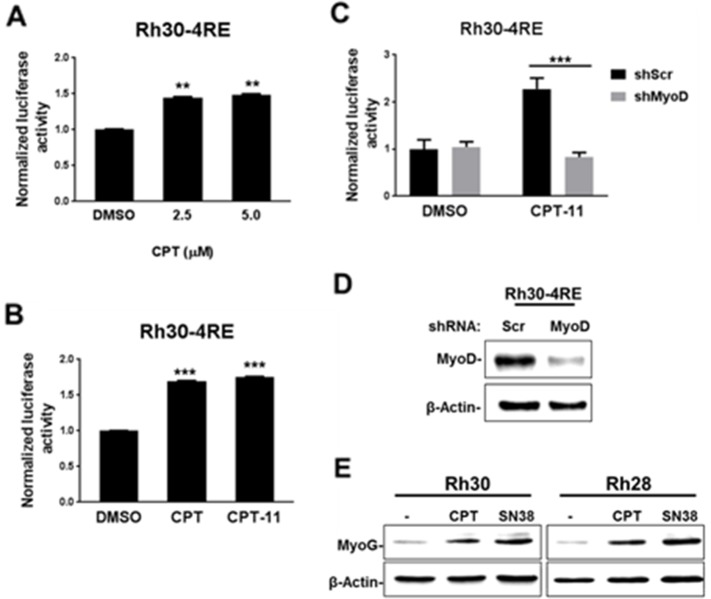
CPT treatment restores MyoD-mediated gene activation in aRMS cells **(A)** Luciferase activity determined from Rh30-4RE cells treated with increasing doses of CPT or DMSO control as indicated for 36 hours in DM. **(B)** Luciferase activity in Rh30-4RE cells treated with 5.0 μM CPT, 5.0 μM CPT-11, or DMSO control as indicated for 36 hours in DM. **(C)** Rh30-4RE reporter cells were treated with CPT-11 or DMSO as in (B), except following expression ofeither scrambled shRNA (shScr) or shMyoD via lentiviral delivery, as indicated. **(D)** MyoD levels assessed in Rh30-4RE cells via immunoblotting following transduction with lentivirus as in (C). **(E)** Low confluency Rh30 and Rh28 cells were treated with 12.0 nM CPT, 5.0 nM SN38, or DMSO control (-) as indicated for 24 hours in DM. MyoG levels were assessed via immunoblotting. β-Actin is used for a loading control. For luciferase activity, values are represented as mean ±SEM (A,B: n=2; C,D: n=3) relative to control cells after normalization to total protein. ^**^ indicates P<0.01; ^***^ indicates P<0.001 relative to DMSO control.

Activation of myogenic gene transcription by MyoD is impaired in aRMS cells [8]. We tested whether CPT activates MyoD-responsive gene transcription in previously described aRMS Rh30-4RE reporter cells [10]. We observed an induction of luciferase activity in CPT-treated Rh30-4RE cells using concentrations similar to those used in C2C12 myoblast cells in Figure 1 (Figure 2A). Additionally, treatment with clinically used CPT analog irinotecan (CPT-11) had the same effect in these reporter cells (Figure 2B) [21, 22]. To test whether this induction of luciferase activity is dependent on MyoD, we depleted MyoD from Rh30-4RE cells and treated them with CPT-11. Indeed, induction of luciferase by CPT-11 was prevented by depletion of MyoD from these reporter cells (Figure 2C, 2D). We next asked whether this activation of MyoD-mediated gene transcription was reflected in its endogenous target gene expression. MyoG is a critical driver of myogenic differentiation, and its upregulation by MyoD is an initiating event in order to execute this process [23, 24]. However, the concentrations of CPT used for the above luciferase assays in aRMS cells were highly cytotoxic relative to comparable treatments with CPT-11 or in murine C2-KMT1A-4RE myoblast cells utilized for the compound screen (Figure 1). Thus we determined the 50% inhibitory concentration (IC50) of CPT in aRMS Rh28 and Rh30 cells in order to use less cytotoxic concentrations (IC50 or sub-IC50) for further experiments (Supplementary Figure 1). We also determined the IC50s of CPT-11 and its active metabolite, SN38 [25], in these cells for reference in subsequent experiments. We then evaluated MyoG levels in aRMS cells following treatment with sub-IC50 doses of CPT or SN38, and found induced MyoG protein levels in both Rh30 and Rh28 cells treated with either CPT or SN38 (Figure 2E). Collectively, the data reveals that treatment of aRMS cells with CPT or tested derivatives leads to restoration of MyoD function and upregulation of MyoG, a critical regulator of myogenic differentiation.

### CPT-11 treatment drives differentiation of aRMS

**Figure 3:**
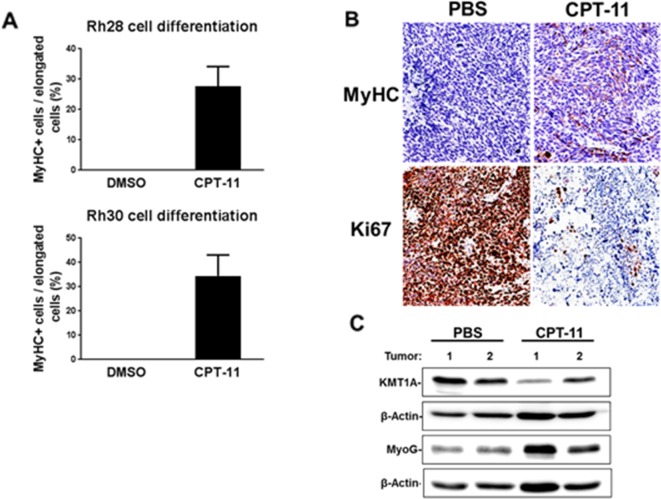
CPT-11 treatment permits differentiation of aRMS cells *in vitro* and *in vivo* **(A)** Quantified MyHC expression in Rh28 and Rh30 cell differentiation following treatment with 2.5 μM CPT-11 or DMSO for 7 days in DM, as indicated. Cells were fixed and subjected to immunofluorescence using MyHC antibodies, and counterstained with DAPI. Data is shown as percentage of MyHC+ cells per total cells showing elongated morphology. Error bars represent ±SEM from at least 3 randomly chosen fields. **(B)** Immunohistochemical staining of Ki67 and MyHC of tumor sections from PBS and CPT-11 treated mice bearing Rh30 xenografts. Mice were treated weekly with either PBS or CPT-11 for 3 weeks. Pictures were taken at 20X magnification. **(C)** Immunoblot analysis of lysates from tumor samples of PBS- or CPT-11-treated mice for KMT1A and MyoG. β-Actin is used for loading control.

CPT-11 was chosen for the following phenotypic studies due to its clinical relevance as part of current chemotherapy protocols to treat RMS [20–22]. Since increased MyoD activity in aRMS cells can lead to terminal differentiation, we asked whether CPT-11 promotes myogenic differentiation in these cells. Hence, we performed immunofluorescence staining of Myosin Heavy Chain (MyHC), a terminal differentiation marker for skeletal muscle [23]. The data shows that CPT-11 treatment leads to MyHC expression in Rh28 and Rh30 cells cultured in DM for 7 days (Figure 3A, Supplementary Figure 2A). Subsequently, the *in vivo* effect of CPT-11 on differentiation was evaluated using an Rh30 aRMS xenograft model. Tumor-bearing mice were treated with CPT-11 or PBS as a control, and tumor volume was measured weekly. Consistent with previous studies treating mice with 10mg/kg CPT-11 weekly [26], a substantial reduction in tumor growth was observed in treated animals (Supplementary Figure 2B). Tumor sections from CPT-11 treated and control mice were subjected to immunohistochemical (IHC) analysis for MyHC, and proliferation marker Ki-67 following experimental endpoints. Indeed, a decrease in Ki-67-positive cells and an increase in MyHC-positive cells were evident in tumor sections from CPT-11 treated mice (Figure 3B). Additionally, lysates from tumor samples were analyzed via immunoblot for KMT1A and MyoG expression. The data shows a loss of KMT1A and induction of MyoG from tumors in mice treated with CPT-11 compared to PBS control (Figure 3C), demonstrating these biochemical changes in therapeutically achievable concentrations in mice. Collectively, these data demonstrate that treatment with CPT-11 leads to the suppression of cell and tumor growth coupled with induction of terminal myogenic differentiation in aRMS.

### CPT treatment leads to downregulation of KMT1A levels in aRMS cells

**Figure 4:**
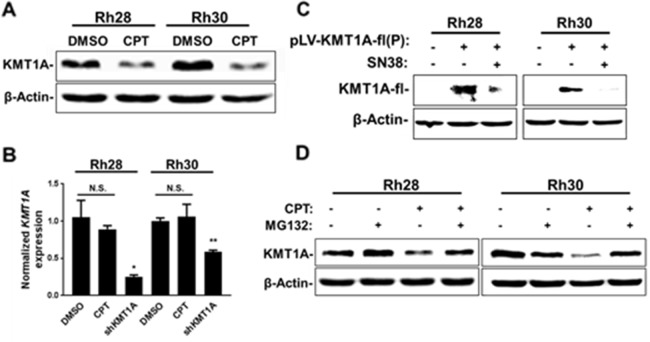
CPT treatment leads to downregulation of KMT1A protein in aRMS cells **(A)** Rh28 and Rh30 cells were treated with 12.0 nM CPT or DMSO control as indicated for 24 hours. KMT1A levels were assessed via immunoblotting. **(B)** Real-time qPCR analysis of KMT1A mRNA levels from Rh28 and Rh30 cells treated with 15.0 nM CPT or DMSO control (-), or transduced with lentivirus expressing shRNA targeting KMT1A as indicated for 24 hours. Data is represented as fold change relative to DMSO treatment following normalization to *ACTB*. Error bars represent ±SEM from reactions performed in triplicate. **(C)** Rh28 and Rh30 cells were transduced with control lentivirus (-) or virus expressing KMT1A-fl cDNA. After several passages, cells were treated with either 2.5 nM SN38 or DMSO control (-) for 48 hours. Ectopic KMT1A-fl levels were then assessed via immunoblot using anti-Flag M2 antibodies. **(D)** Rh28 and Rh30 cells were pre-treated with either 100.0 nM MG132 or DMSO control (-) for 1 hour before addition of 30.0 nM CPT as indicated. After 24 hours, cells were collected and KMT1A levels were assessed via immunoblotting. For all immunoblot analysis, β-Actin is used for loading controls. ^*^ indicates P<0.05; ^**^ indicates P<0.01.

Our observations in CPT and derivative-treated aRMS cells mimic the effects seen following KMT1A depletion [10]. Moreover, CPT was selected for this study based on overcoming KMT1A-mediated suppression of MyoD activity in myoblasts. This led us to determine whether the effect of CPT on MyoD activity occurs through KMT1A in aRMS cells. Hence, we evaluated MyoD-dependent gene activation by measuring luciferase activity in Rh30-4RE cells and MyoG expression in Rh30 cells following knockdown of KMT1A and subsequent treatment with CPT-11 or CPT. The results showed that CPT-11 and CPT treatment have no additive effect on reporter luciferase activity and MyoG expression in KMT1A-depleted aRMS cells, respectively (Supplementary Figure 3A). This indicates that this restoration of MyoD activity by CPT/CPT-11 is through KMT1A. Thus, we evaluated KMT1A levels in aRMS cells following treatment with a sub-IC50 dose of CPT. The data showed decreased protein levels of KMT1A in CPT-treated Rh28 and Rh30 cells (Figure 4A). Rh30 cells treated with CPT-11 in a dose-dependent manner also showed loss of KMT1A (Supplementary Figure 3B). Subsequently, we evaluated whether this loss of KMT1A expression occurs at the mRNA level, using cells genetically depleted of KMT1A as a positive control. The data showed that treatment of Rh28 and Rh30 cells treated with CPT results in no changes in KMT1A mRNA levels (Figure 4B). Using a different experimental approach, we also validated that SN38 downregulates KMT1A at the protein level by overexpressing Flag epitope-tagged KMT1A (KMT1A-fl) in Rh28 and Rh30, and treating these cells with SN38. The data shows that SN38 treatment downregulated the ectopically expressed KMT1A-fl protein (Figure 4C). Together, these results indicate that loss of KMT1A occurs at the protein level in CPT and CPT derivative-treated aRMS cells. Since KMT1A is regulated by proteasome-mediated degradation [27], we tested whether treatment of aRMS cells with CPT results in proteasome-mediated degradation of KMT1A. Results show that simultaneous treatment of Rh28 and Rh30 cells with proteasome inhibitor MG132 and CPT recovers the loss of KMT1A protein (Figure 4D). Collectively, these data show that CPT treatment of aRMS cells results in downregulation of KMT1A protein through proteasome-dependent degradation.

### Downregulation of KMT1A by CPT occurs independently of topoisomerase I cleavage complexes

**Figure 5:**
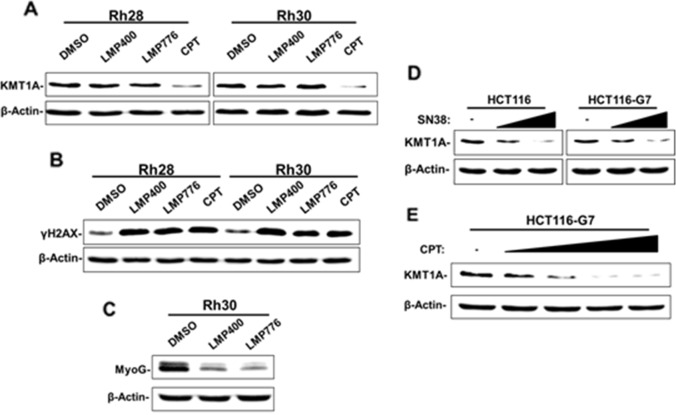
Downregulation of KMT1A by CPT is independent of TOP1-DNA cleavage complex **(A)** Rh28 cells were treated with 63.0 nM LMP400, 17.0 nM LMP776, 30.0 nM CPT, or DMSO control as indicated for 24 hours. Rh30 cells were treated with 53.0 nM LMP400, 13.0 nM LMP776, 38.0 nM CPT, or DMSO control as indicated for 24 hours. KMT1A levels were then assessed by immunoblotting. **(B)** Rh28 and Rh30 cells were treated as in (A) and were subjected to immunoblot analysis to determine levels of γH2AX. Total H2A is used as additional loading control. **(C)** Rh30 cells were treated with LMP400, LMP776, or DMSO control as in (A), and MyoG levels were assessed via immunoblotting. **(D)** HCT116 and HCT116-G7 cells were treated with SN38 (2.5 nM and 5.0 nM) or DMSO control (-) as indicated for 48 hours. KMT1A levels were then assessed by immunoblotting. **(E)** HCT116-G7 cells were treated with increasing doses of CPT (5.0 nM, 10.0 nM, 25.0 nM, and 50.0 nM) or DMSO control (-) as indicated for 48 hours. KMT1A levels were then assessed by immunoblotting. For all immunoblot analysis, β-Actin is used for loading controls.

CPT generates DNA damage through TOP1-DNA cleavage complexes [18]. We evaluated whether loss of KMT1A results from this mechanism in CPT-treated cells. For this purpose, we utilized two non-CPT synthetic compounds, indotecan (LMP400) and indimitecan (LMP776), which have the same mechanism of action against TOP1 [28, 29]. We first determined the IC50 concentrations of LMP400 and LMP776 in Rh28 and Rh30 cells in the same manner as we determined for CPT (Supplementary Figure 4). Subsequently, these cells were treated with CPT, LMP400, or LMP776 at their respective IC50s for one day and evaluated levels of KMT1A and phosphorylated H2AX (γH2AX), a DNA damage biomarker [30]. The results showed that CPT, but not LMP400 or LMP776, decreased KMT1A protein levels in treated cells (Figure 5A). In contrast, levels of γH2AX were similar in both cell lines treated with these compounds (Figure 5B). Under these conditions, phase-contrast microscopy results also confirmed that the treatments used were largely non-cytotoxic (Supplementary Figure 5A). In contrast to MyoD activity-induced MyoG following CPT treatment (Figure 2E), the LMP compounds fail to increase MyoG expression but rather decrease it in Rh30 cells (Figure 5C). This decrease in MyoG may be a consequence of more acute cytotoxicity and sustained TOP1-DNA cleavage complexes caused by LMP compounds compared to CPT [28, 29], and is currently under investigation. In order to confirm that the lack of KMT1A downregulation by LMP compounds was not restricted to the conditions used, we also tested whether LMP400 treatment depletes KMT1A protein from Rh30 cells in a dose-dependent manner, with CPT as a positive control. The data confirms that KMT1A is not downregulated in LMP400-treated Rh30 cells at a range of concentrations (Supplementary Figure 5B). These results imply that loss of KMT1A protein following CPT treatment is independent of treatment-induced DNA damage. To further investigate this, we utilized a cell system harboring SN38-resistant TOP1. Derived from parental HCT116 colon cancer cells, HCT116-G7 is a SN38-resistant clonal cell line with mutations in each *TOP1* allele [31]. Treatment with increasing doses of SN38 confirmed resistance of HCT116-G7 cells, as revealed by a lack of DNA-damage induced γH2AX relative to HCT116 (Supplementary Figure 6A). However, both cell lines showed dose-dependent loss of KMT1A protein following SN38 treatment (Figure 5D). We asked whether the loss of KMT1A in SN38-resistant HCT116-G7 cells could be recapitulated with CPT treatment. Similarly to SN38, these cells were resistant to CPT treatment relative to HCT116 at a highly cytotoxic dose (Supplementary Figure 6B). However, KMT1A was downregulated from HCT116-G7 cells treated with lower concentrations of CPT (Figure 5E). Taken together, these data uncover that downregulation of KMT1A by CPT in cells occurs independently of the well-established DNA damage-inducing interaction with TOP1.

### CPT derivatives inhibit KMT1A enzymatic activity *in vitro*

**Figure 6:**
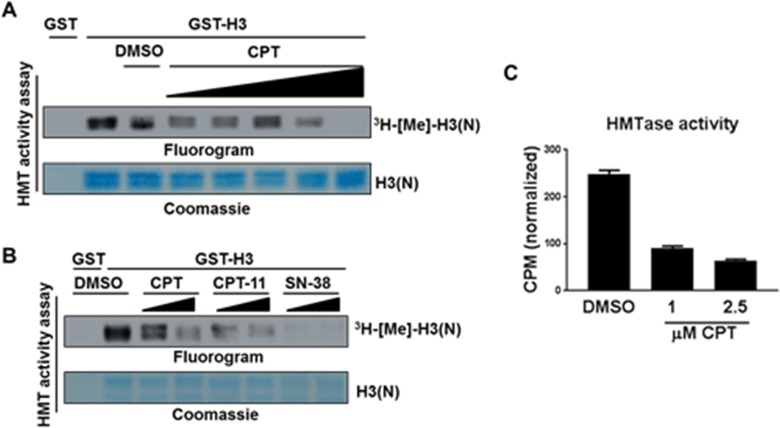
CPT inhibits KMT1A enzymatic activity in an *in vitro* reconstituted system **(A)** HMTase assay with GST-KMT1A, [^3^H]-SAM cofactor, and either GST or GST-H3(N) substrate as indicated. Reactions contained either increasing concentrations of CPT (0.25 μM, 0.5 μM, 1 μM, 4 μM, and 5 μM) or maximum volume of DMSO, as indicated. H3(N) was visualized by coomassie staining, and ^3^H-[Me]-H3(N) by autoradiography. **(B)** Same as (A) except reactions were carried out using 2.5 μM or 5.0 μM of CPT, CPT-11 and SN38 as indicated. **(C)** HMTase assay with reactions carried out similarly as in (A), except samples were precipitated on filter paper, washed, dried, and subjected to scintillation counting. Counts per minute (CPM) were normalized by subtraction of background signal as measured by a control reaction lacking enzyme. Error bars represent ±SEM from reactions performed in duplicate.

Treatment of cells with CPT as well as its derivatives CPT-11 and SN38 influences KMT1A independently of DNA damage induction, which raises the possibility that CPT can modulate KMT1A activity. KMT1A is best known for catalyzing tri-methylation of histone H3 lysine 9 (H3K9me3) [32]. Thus, we examined the effect of CPT on KMT1A activity in an *in vitro* histone methyltransferase (HMTase) assay. This HMTase assay was performed using purified KMT1A, H3 as a substrate, and ^3^H radiolabeled S-Adenosylmethionine (SAM) as a cofactor in the presence or absence of increasing doses of CPT. The data shows dose-dependent inhibition of KMT1A methyltransferase activity in the presence of CPT (Figure 6A). Furthermore, a subsequent experiment showed that CPT-11 and SN38 have similar dose-dependent inhibitory effects on KMT1A methyltransferase activity in this assay system (Figure 6B). To confirm this observation, we sought to recapitulate this experiment using recombinant KMT1A from a commercial source with HMTase activity measured via a different method. Indeed, KMT1A activity measured by precipitation of reaction proteins followed by scintillation counting also revealed a substantial inhibition of KMT1A in the presence of CPT compared to DMSO control (Figure 6C). Collectively, these data demonstrate that CPT can directly inhibit KMT1A activity *in vitro*.

## DISCUSSION

Our data reveals that treatment with CPT or select CPT derivatives (CPT-11 and SN38) promotes MyoD activity and a differentiated myogenic phenotype in aRMS cells. The results also show that CPT-11 treatment results in differentiation of aRMS in an *in vivo* xenograft model. While this effect is similar to the observed phenotype following genetic depletion of KMT1A [10], it is notable that a previous report demonstrated that treatment with CPT suppresses PAX3-FOXO1 expression in aRMS cells, although the mechanism remains unclear [33]. It has separately been reported that depletion of PAX3-FOXO1 from aRMS cells induces myogenic differentiation [34]. As both aRMS cell lines used in this study are PAX3-FOXO1-positive, this raises the possibility that loss of PAX3-FOXO1 contributes to the differentiated phenotype we observed in CPT-treated aRMS cells. However, genetic depletion of PAX3-FOXO1 also results in a loss of MyoD expression, as MyoD is a PAX3-FOXO1 target gene [34, 35]. This contrasts to genetic depletion of KMT1A, which results in promotion of MyoD activity similarly to our observations in CPT-treated aRMS cells [10]. Additionally, we found that CPT does not influence myogenic gene activation in aRMS cells following knockdown of KMT1A. Thus, our results support a model wherein KMT1A suppression is primarily responsible for MyoG induction and myogenic differentiation in CPT-treated aRMS cells.

We probed the mechanism by which KMT1A protein is depleted from CPT-treated cells. In order to address whether CPT-induced TOP1-DNA cleavage complexes were involved in this loss of KMT1A in aRMS cells, we utilized two non-CPT compounds LMP400 and LMP776. These compounds were identified for their ability to target TOP1 in the same manner as CPT, and several independent lines of evidence demonstrate this activity by the LMP compounds [28, 29, 36]. We also utilized HCT116-G7 colon cancer cells, which lack wild-type TOP1 and are resistant to cytotoxic effects CPT derivatives [31, 37]. Collectively, our data represents compelling evidence that CPT downregulates KMT1A independently of TOP1-DNA cleavage complexes. To our knowledge, this is the first report which demonstrates such an activity of CPT and its analogues CPT-11 and SN38 against KMT1A. Since CPT is a naturally occurring compound among the most extensively studied in biology, this finding may have significant impact on future research and development of CPT analogues for anticancer agents. In this regard, growing evidence indicates that novel CPT analogues often have biological activity independent of their target TOP1. For example, the CPT analogue FL118 shows antitumor activity in TOP1-low/negative cancer [38]. Similarly, another report demonstrated that a CPT analogue (O2-16) that is inactive against TOP1 showed broad antiviral HIV-1 activity [39]. These studies plus our current report indicate a new trend for reconsidering the mechanistic potential of CPT and its derivatives.

We further observed direct inhibition of KMT1A activity by CPT, CPT-11, and SN38 using an *in vitro* reconstituted system. Notably, the concentration range of CPT used in this KMT1A methyltransferase assay is similar to that first used to establish its effect on TOP1 [18]. CPT-11 also showed inhibitory effect on KMT1A activity in this concentration range. This differs from its established mechanism with TOP1, which requires hydrolysis of CPT-11 by carboxylesterases to form SN38 *in vivo* [40]. CPT-11 shows marginal effect on TOP1 *in vitro* at concentrations below 1mM [25]. Therefore, the inhibitory effect of CPT-11 on KMT1A activity in our assay provides strong evidence for a novel biochemical activity of CPT and its tested derivatives CPT-11 and SN38. Importantly, our data does not firmly establish whether the enzymatic inhibition of KMT1A by CPT observed *in vitro* occurs in cells to result in the observed degradation of KMT1A. However, in some contexts KMT1A exists in cells as part of a multimeric methyltransferase complex that contributes to its stability, and furthermore KMT1A is a substrate for ubiquitination by the E3-ubiquitin ligase MDM2 [27, 41]. Our data demonstrate that CPT treatment inhibits KMT1A enzymatic activity *in vitro* while leading to degradation of the protein in cells. Collectively, we propose that CPT treatment influences the binding of KMT1A to protein complexes, destabilizing the KMT1A protein and promoting degradation via the proteasome. *In vitro*, this influence of KMT1A structure by CPT manifests as a loss of enzymatic activity, but cannot lead to protein degradation due to a lack of cellular factors. Future studies will be aimed at elucidating the precise mechanism by which KMT1A protein is lost in CPT-treated cells. Currently, CPT-11 is part of the VAC/VI and VITA chemotherapy regimens for treating high-risk RMS [20–22], and showed activity as a single agent in heavily pretreated patients with relapsed RMS [42]. However, it is distinctly possible that a CPT derivative with stronger anti-KMT1A activity than CPT-11 could be more efficacious in treating aRMS. As there are numerous CPT derivatives in various stages of preclinical and clinical development beyond those used in this work [43], further study to identify which of those compounds has anti-KMT1A activity, and how efficiently they can drive differentiation in aRMS cells, is warranted. Concurrently, it will be important to better understand the anti-KMT1A mechanism of CPT in cells, its link to the enzymatic inhibition *in vitro*, and evaluate its implications in a broad scientific landscape.

In conclusion, our results uncover that the well-studied anti-cancer TOP1 poison CPT can overcome KMT1A-mediated repression of MyoD activity and induce a differentiated myogenic phenotype in aRMS cells. Our results also indicate that these effects of CPT on aRMS cells are at least partially from suppression of KMT1A by CPT and its analogues. Furthermore, the inhibitory effect of CPT on KMT1A occurs independently of CPT-induced DNA damage response. As CPT derivative CPT-11 mimics these effects and is currently used to treat patients, this study is highly clinically relevant to aRMS.

## MATERIALS AND METHODS

### Cell culture, lentiviral vectors, and lentiviral transduction

Human aRMS cell lines Rh28 and Rh30 were provided by Peter Houghton (Greehey CCRI), and authenticated by confirming PAX3-FOXO1 expression. 293FT viral packaging cells were purchased from Invitrogen, and transfected with necessary viral vectors using PEI (Polybiosciences #24765) according to manufacturer's protocol. In brief, DNA plasmids were mixed with PEI at a 1:3 ratio (m/m) in serum-free D-MEM for 20-30 minutes before being added dropwise to 293FT cells and cultured overnight. Lentiviral particles were harvested 48 hours post-transfection. Colon cancer HCT116 cells were previously provided by Bert Vogelstein(Johns Hopkins) [44]. TOP1-mutant HCT116-G7 colon cancer cells were provided by Celine Gongora and Maguy Del Rio [31, 37]. Except C2C12 myoblasts (cultured in 20% FBS), all cells were cultured in D-MEM (Corning) containing 10% FBS (growth medium, GM), or D-MEM containing 2% horse serum and 10 μg/mL insulin (differentiation medium, DM) where indicated. All media contained 1x Antibiotic-Antimycotic solution (Gibco #15240) KMT1A or MyoD levels were manipulated in cells by transduction with lentivirus expressing shRNA targeting MyoD or KMT1A, or expressing a Flag epitope-tagged KMT1A cDNA. All viral vectors have been describedpreviously [19, 10]. Oligo sequences for shRNA vectors are as follows: MyoD, 5′ CCGCCAGGATATGGAGCTA-3′; KMT1A, 5′-GGGTATCCGATATGACCTC-3′. Stable KMT1A-overexpressing C2C12 reporter cells containing MyoD-responsive 4RE luciferase gene (C2-KMT1A-4RE) have been described previously [14], as have Rh30-4RE reporter cells [10]. All cells were routinely tested for mycoplasma, and cultured at 37°C and 5% CO_2_ in a humidified atmosphere.

### Chemical screen and compounds

Chemical screen using C2-KMT1A-4RE reporter cells was standardized in a 96 well format plating 5 × 10^3^ cells per well. Cells were exposed to the Spectrum Collection compound library (MicroSource Discovery System) of pharmacological compounds at 10 μM in DMSO for 36 hours in DM using JANUS Automated Liquid Handling Workstation and PlateStak Automated Microplate System (PerkinElmer). Luciferase activity was determined using Envision 2103 Multilabel Reader (PerkinElmer). CPT was purchased from Sigma and stocks were dissolved in DMSO. CPT-11 was purchased from LC Laboratories (I-4122), and SN38 was received as a gift from the laboratory of Katerina Gurova (Roswell Park) where it was purchased from Tocris (#2684). CPT-11 was consistently used at higher concentrations relative to CPT and SN38, as it is a pro-drug and requires higher doses for cytotoxic effects [45]. LMP400 and LMP776 were obtained from the NCI Developmental Therapeutics Program Open Chemicals Repository and dissolved in DMSO. All compounds used were stored at −20°C.

### Immunoblotting, immunofluorescence, and antibodies

Preparation of cell extracts and immunoblot analyses were carried out as described previously [46]. Immunofluorescence was carried out as described previously [10], with VECTASHIELD mounting medium (Vector Labs) used to counterstain with DAPI. Antibodies used were β-Actin-peroxidase (Sigma A3854), Flag-M2-HRP (Sigma A8592), MyoD (Santa Cruz sc-304), MyoG (BD Pharmingen 556358), KMT1A (Cell Signaling 8729), total histone H2A (Cell Signaling L88A6), phospho-H2AX (Ser 139) (Cell Signaling 20E3), and MyHC (Developmental Studies Hybridoma Bank, MF-20). Images were generated using the FluorChem HD2 (Alpha Innotech) or ChemiDoc Touch (Bio-Rad) imaging systems, which produce high contrast images and can identify over-exposed protein bands.

### Luciferase reporter and cell viability assays

Reporter luciferase activity was determined as described previously using a commercial assay system (Promega) [46], except luciferase from stable reporter cells was normalized to protein concentration of the lysate. Cell viability assays were performed by fixing and staining cells with a solution containing 1% methylene blue and 50% methanol in PBS. After drying, the dye was extracted using 1% SDS in PBS. Luciferase activity and methylene blue absorbance were measured using a VICTOR Multilabel Plate Reader (Perkin Elmer).

### Gene expression analysis

RNA was extracted from cells using TRIzol Reagent (Sigma). cDNA synthesis was performed using SuperScript III First-Strand Synthesis System (Invitrogen). Quantitative real-time PCR was performed using FastStart Universal SYBR Green Master Mix with ROX dye (Roche) and a QuantStudio 6 Real-Time PCR system (ThermoFisher Scientific). All expression changes were quantified using the delta-delta C_T_ method with reactions performed as technical triplicates. Data is representative of multiple independent experiments. Primers for *ACTB* and *KMT1A* are as follows: ACTB-F: 5′-CACACTGTGCCCATCTACG-3′; ACTB-R: 5′-TGC TTGCTGATCCACATC-3′; KMT1A-F: 5′-GCACAAG TTTGCCTACAA-3′, KMT1A-R: 5′CCAGGTCAAAG AGGTAGGTG-3′.

### Histone methyltransferase (HMTase) assay

Purification of recombinant GST-KMT1A, GST-H3(N) and HMTase assays were performed similarly as previously described [14, 47]. For purification of GST proteins, fresh 250 mL cultures of *E. coli* containing inducible GST-KMT1A or GST-H3(N) plasmids were grown to O.D. 0.5-0.6 and induced with 0.1 mM IPTG for 3 hours at 37°C. Following lysis, 10 mg of extract was incubated with 500 μL of glutathione agarose beads in a 50% slurry. Beads were then washed and eluted using reduced glutathione (Sigma, G4251). Protein purity was verified by resolving 2 μg of protein via SDS-PAGE and staining with Coomassie blue. Histone methylation was assayed using 2.5 μg of purified recombinant GST-KMT1A with 2.5 μg of purified GST-H3(N) as a substrate. Each reaction contained 50 mM Tris-Cl (pH 8.5), 20 mM KCl, 10 mM MgCl_2_, 10 mM β-mercaptoethanol, and 0.5 μCi of [^3^H]-S-adenosyl-L-methionine (SAM) (Perkin Elmer) in 25 μL reactions. Samples were incubated in the presence of chemicals or DMSO as a negative control for 2 hours at 30°C. Reactions were terminated, resolved using SDS-PAGE, and visualized via autoradiography. For quantitative analysis of HMTase activity, GST-KMT1A was purchased from Abcam (ab80289). Reactions were carried out similarly as above, except 0.5 μg of purified enzyme was used. Following the 2 hour incubation, reaction mixtures were precipitated by being spotted onto Whatman P81 filter paper and allowed to dry for approximately 1 hour. Samples were then washed 3 times with 1M NaHCO_3_ (pH 9) for 20 minutes per wash, and allowed to dry overnight on chromatography paper. Radioactivity was quantified by scintillation counting.

### Animal model

Animal procedures were performed in accordance with IACUC-approved protocol as previously described [48]. A total of 5 × 10^6^ Rh30 cells were suspended in 100 μL PBS and injected into each flank of six 6-10 week old athymic female NOG mice (Taconic). Mice were monitored until tumors were approximately 50 mm^3^, and split into treatment and control groups. Mice were administered either CPT-11 (10 mg/kg) or PBS via tail vein injection weekly, and tumor growth was measured weekly by digital caliper. At the end-point of the experiment (when a single tumor reached ~1000 mm^3^ in volume), the mice were sacrificed and the tumors harvested for immunohistochemistry (IHC) staining, which was carried out using Ki-67 or MyHC (MF20) antibodies as previously described by the Pathology Network Shared Resource at Roswell Park [48].

### Statistical analysis

Data were evaluated using the unpaired Student's *t*-test and GraphPad Prism version 7. P < 0.05 was considered statistically significant. IC50 values were calculated using GraphPad Prism version 7, with DMSO control considered 100% cell viability and the greatest cytotoxicity measured considered 0% cell viability.

## SUPPLEMENTARY MATERIALS FIGURES AND TABLE


