# Supplementary material for: Camptothecin exhibits topoisomerase1-independent KMT1A suppression and myogenic differentiation in alveolar rhabdomyosarcoma cells
Source: Oncotarget. 2018 May 25;9(40):25796–807. doi: 10.18632/oncotarget.25376 (PMC5995248; doi:10.18632/oncotarget.25376)
Supplement: Supplementary file 1 [file oncotarget-09-25796-s001.pdf]

Camptothecin exhibits topoisomerase1-independent KMT1A suppression and myogenic differentiation in alveolar rhabdomyosarcoma cells

SUPPLEMENTARY MATERIALS

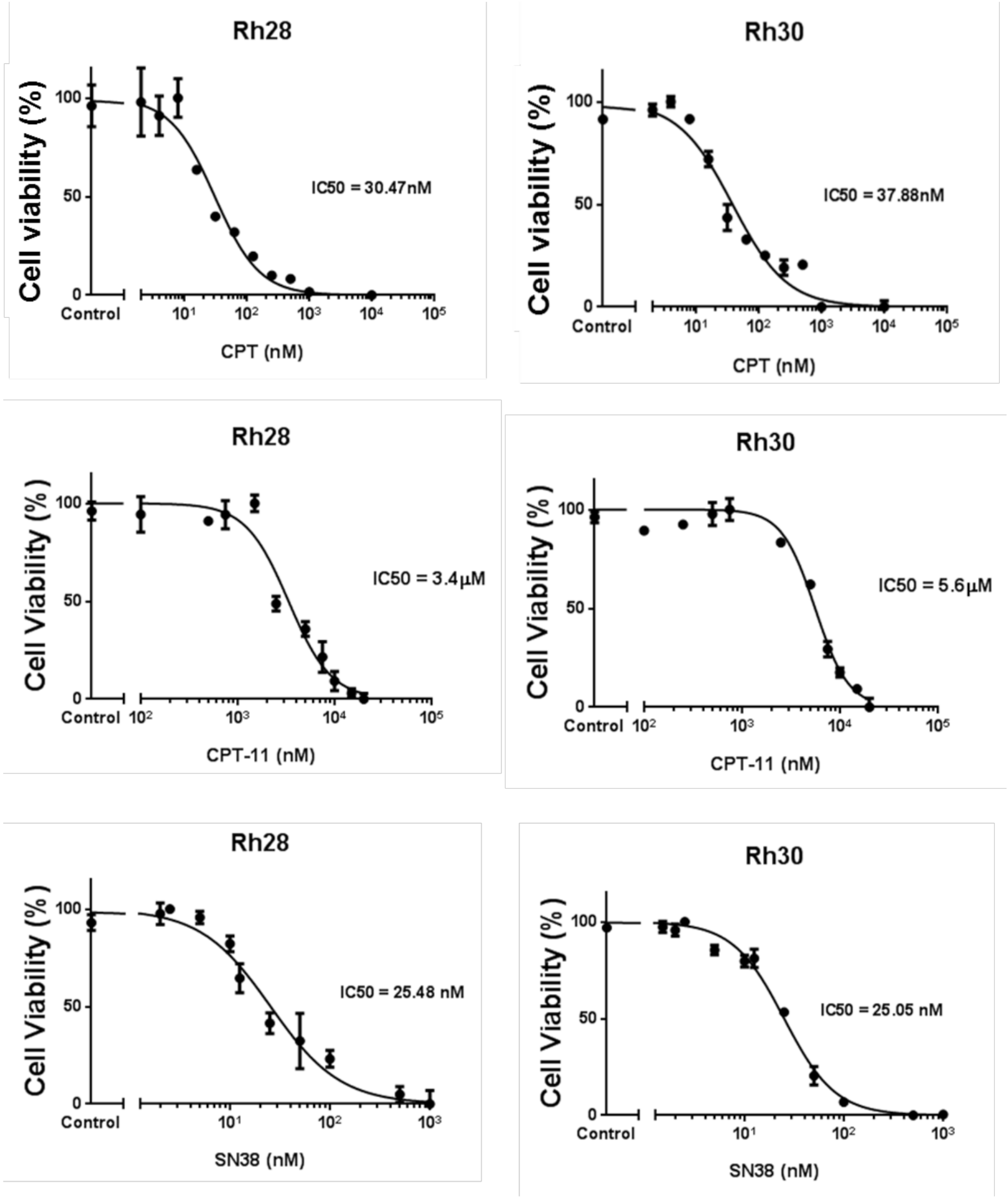

Supplementary Figure 1: IC50 values of CPT and select derivatives in aRMS cells. Rh28 and Rh30 were treated with varying doses of CPT, CPT-11, or SN38 as indicated for 48 hours. Cells were fixed and stained with methylene blue, and cell viability was quantified following extraction of the dye. Data is represented as the mean ±SEM (n=2).

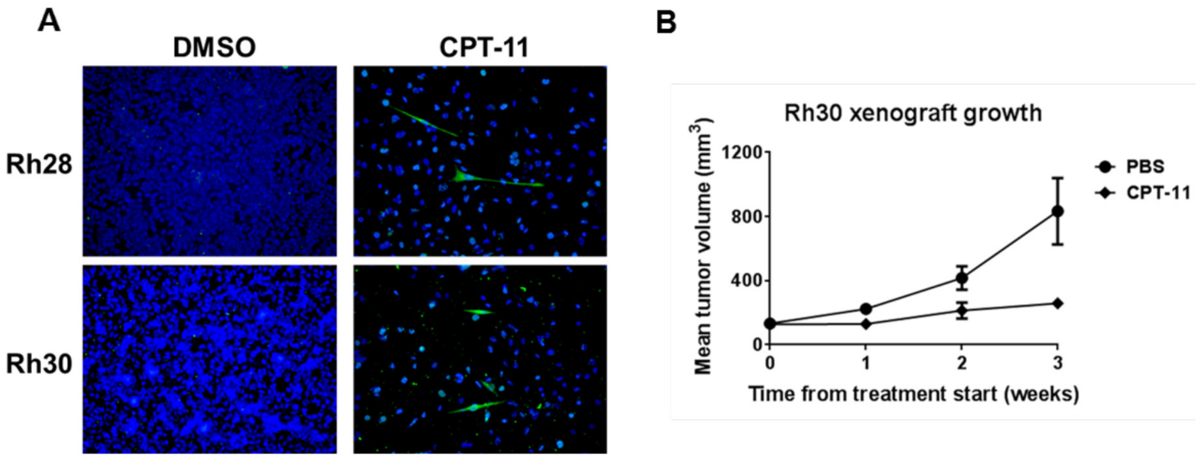

Supplementary Figure 2: CPT-11 treatment restores differentiation in aRMS cells. (A) Micrographs of aRMS cells from Figure 4A subjected to immunostaining for MyHC (green) and counterstained with DAPI. **(B)** Volume of Rh30 xenograft tumors analyzed in Figure 4B, C. Volume was measured weekly, and data is represented as mean tumor volume ±SEM (n≥4 tumors).

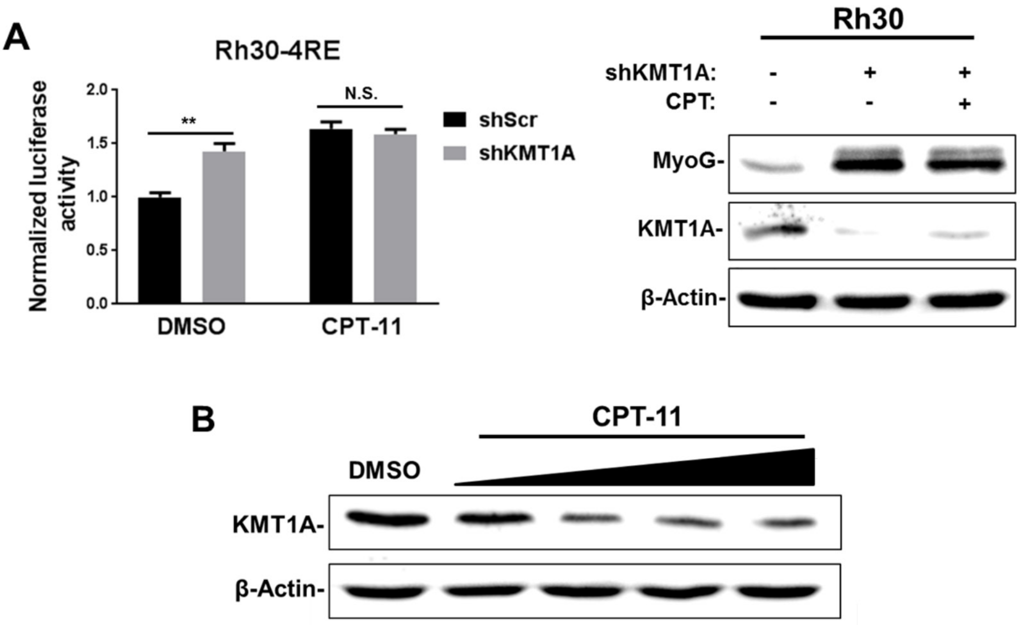

Supplementary Figure 3: Activation of MyoD by CPT occurs through KMT1A in aRMS cells. (A) (*Left*) Luciferase activity from Rh30-4RE cells treated with DMSO or CPT-11 following transduction with lentivirus as in Figure 2, except using lentivirus expressing an shRNA targeting KMT1A. Values are represented as mean ±SEM (n=3). N.S. indicates P>0.05. (*Right*) Rh30 cells were treated with 30.0 nM CPT or DMSO control (-) for 24 hours in DM following transduction with lenti-virus as in Figure 2. MyoG and KMT1A levels were assessed via immunobloting. β-actin is used as a loading control. ** indicates P<0.01. **(B)** Rh30 cells were treated with DMSO or increasing doses of CPT-11 (1.0, 2.5, 5.0, 7.5 μM) for 24 hours. KMT1A levels were then assessed via immunobloting. β-Actin is used as a loading control.

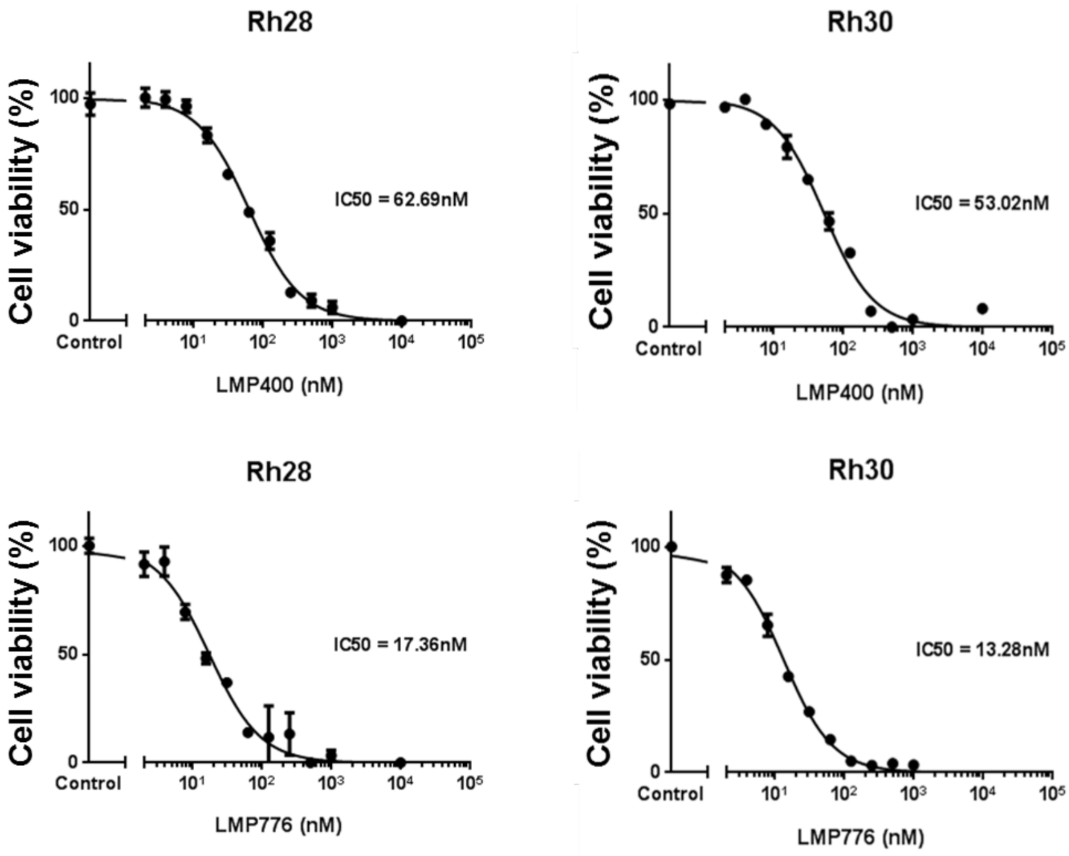

Supplementary Figure 4: IC50 values of LMP400 and LMP776 in aRMS cells. Rh28 and Rh30 were treated with varying doses of LMP400 and LMP776 as indicated for 48 hours. Cells were fixed and stained with methylene blue, and cell viability was quantified following extraction of the dye. Data is represented as the mean ±SEM (n=2).

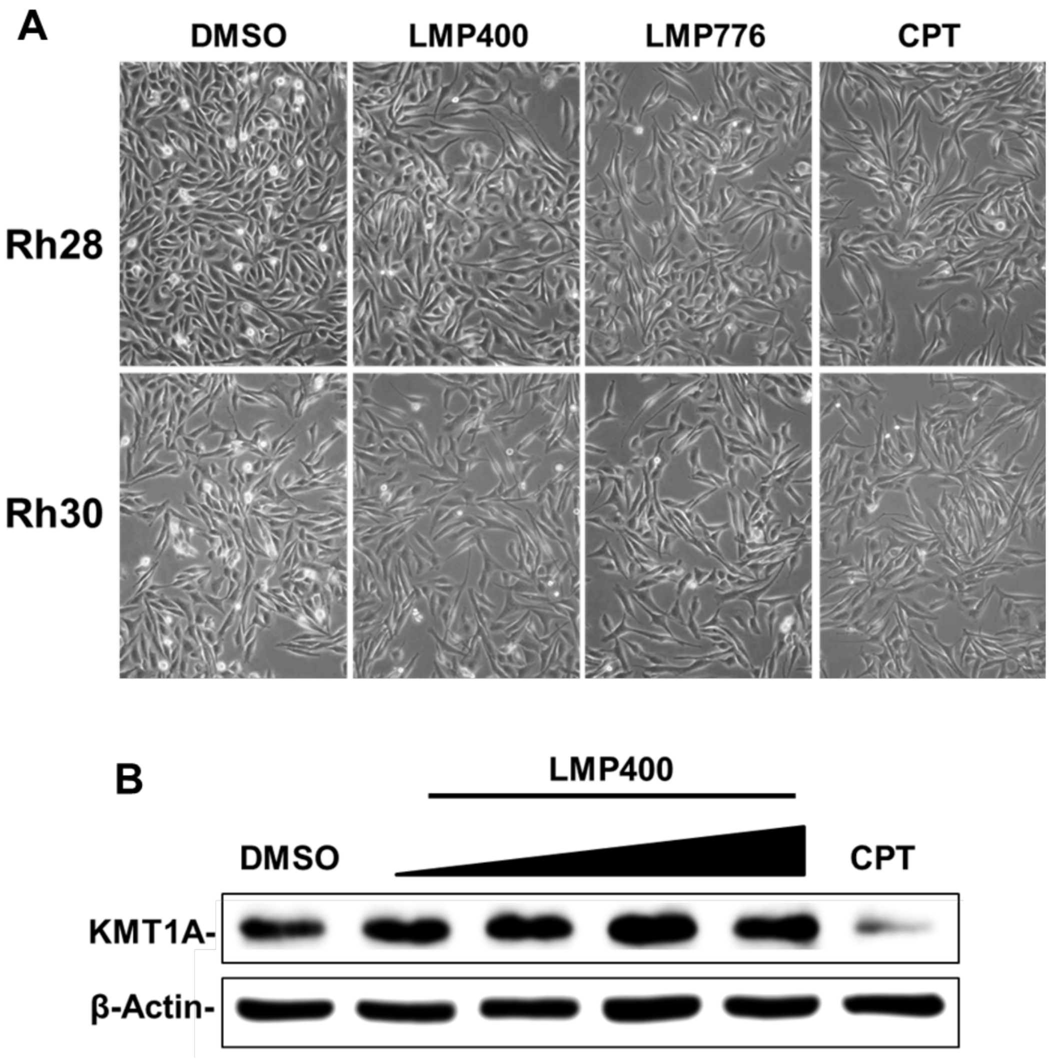

Supplementary Figure 5: Analysis of cytotoxicity of CPT, LMP400, and LMP776 in aRMS cells. **(A)** Phase-contrast micrographs of Rh28 and Rh30 cells treated as in Figure 6A. Pictures were taken at a 40X magnification. **(B)** Rh30 cells were treated with increasing doses of LMP400 (25.0 nM, 50.0 nM, 75.0 nM, and 100.0 nM), 30.0 nM CPT, or DMSO control as indicated for 24 hours. KMT1A levels were then assessed by immunoblotting. β-Actin are used as loading control.

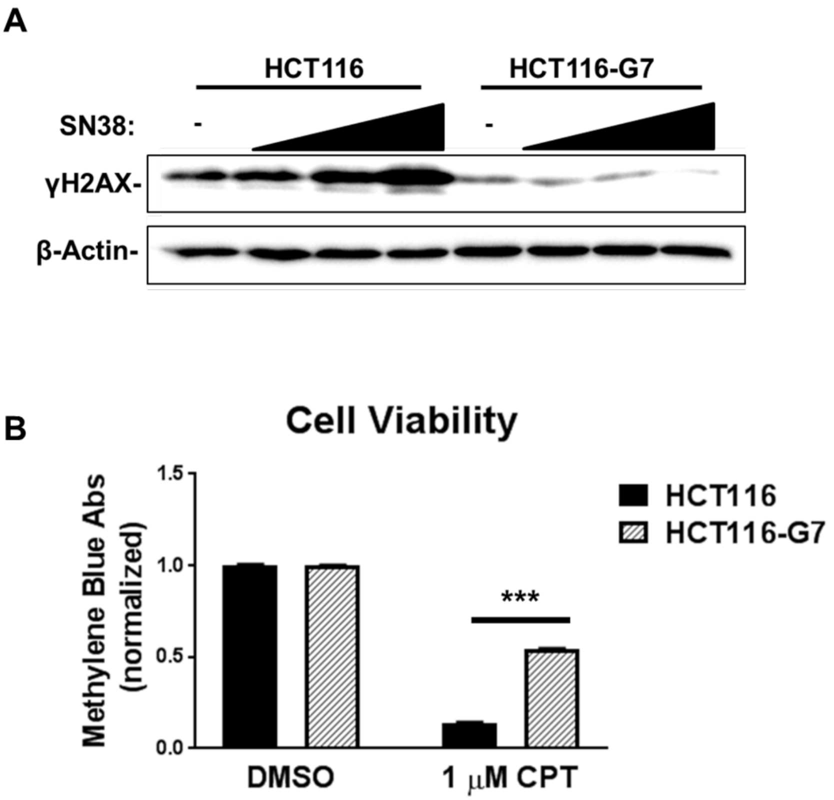

Supplementary Figure 6: HCT116-G7 cells are resistant to DNA damage by SN38 and CPT. **(A)** HCT116 and HCT116-G7 cells were treated with increasing doses of SN38 (2.5 nM, 5.0 nM, 10.0 nM) or DMSO control (-) for 48 hours. γH2AX levels were determined by immunoblot. β-Actin is used as loading control. **(B)** HCT116 and HCT116-G7 cells were treated with DMSO or CPT as indicated for 4 days. Cells were fixed and stained with methylene blue, and cell viability was quantified following extraction of the dye. Data is represented as the mean ±SEM (n=3). *** indicates P<0.001.

Supplementary Table 1: Hit compounds confirmed by secondary screening. C2-KMT1A-4RE cells were treated with increasing concentrations of each primary hit identified in Figure 1B. Of these, 15 compounds which demonstrated >2 fold induction of luciferase were considered confirmed hits and are listed here.

Hits confirmed by secondary screening	PubChem ID#
OXIBENDAZOLE	4622
TOPOTECAN HYDROCHLORIDE	60699
EDOXUDINE	66377
10-HYDROXYCAMPTOTHECIN	97226
CAMPTOTHECIN	24360
EUPARIN	119039
DECAHYDROGABOGIC ACID	5149276
PINOSYLVIN METHYL ETHER	5281719
beta-PELTATIN	92122
APIGENIN TRIACETATE	18721
4’-HYDROXYFLAVANONE	165506
GENISTEIN	5280961
DERRUSNIN	17155307
PRIMULETIN	68112
ALBENDAZOLE	2082
